# The role of environmental tobacco exposure and *Helicobacter pylori* infection in the risk of chronic tonsillitis in children: O papel da exposição ambiental do tabaco e infecção pelo *Helicobacter pylori* no risco de amigdalite crônica em crianças

**DOI:** 10.1590/1516-3180.2016.023602102016

**Published:** 2017-01-05

**Authors:** Chen Li’e, Che Juan, Jiang Dongying, Feng Guiling, Zheng Tihua, Wang Yanfei

**Affiliations:** I MD. Attending Physician, Department of Otorhinolaryngology, Binzhou Medical University Hospital, Binzhou, Shandong, China.; II MD. Attending Physician, College of Special Education, Binzhou Medical University, Yantai, Shandong, China.

**Keywords:** Helicobacter pylori, Tonsillitis, Tobacco smoke pollution, Children, Infection

## Abstract

**CONTEXT AND OBJECTIVE:**

*Helicobacter pylori* (*H. pylori*) is a chronic infectious pathogen with high prevalence. This study investigated the interaction between environmental tobacco exposure and *H. pylori* infection on the incidence of chronic tonsillitis in Chinese children.

**DESIGN AND SETTING:**

Cross-sectional study performed in an outpatient clinic in China.

**METHODS:**

Pediatric patients with chronic tonsillitis were enrolled. *H. pylori* infection was determined according to the presence of H. pylori CagA IgG antibodies. Serum cotinine levels and environmental tobacco smoke (ETS) exposure were determined for all participants.

**RESULTS:**

There was no significant difference in *H. pylori* infection between the children with chronic tonsillitis and children free of disease, but there was a significant difference in ETS between the two groups (P = 0.011). We next studied the association between ETS and chronic tonsillitis based on *H. pylori* infection status. In the patients with *H. pylori* infection, there was a significant difference in ETS distribution between the chronic tonsillitis and control groups (P = 0.022). Taking the participants without ETS as the reference, multivariate logistic regression analysis showed that those with high ETS had higher susceptibility to chronic tonsillitis (adjusted OR = 2.33; 95% CI: 1.67-3.25; adjusted P < 0.001). However, among those without *H. pylori* infection, ETS did not predispose towards chronic tonsillitis.

**CONCLUSION:**

Our findings suggest that tobacco exposure should be a putative mediator risk factor to chronic tonsillitis among children with *H. pylori* infection.

## RESUMO

**CONTEXTO E OBJETIVO:**

*Helicobacter pylori* (*H.* *pylori*) é um patógeno infeccioso crônico com alta prevalência. Este estudo investigou a interação entre exposição à fumaça ambiental do tabaco (FAT) e infecção pelo *H.* *pylori* sobre a incidência de amigdalite crônica em crianças chinesas.

**TIPO DE ESTUDO E LOCAL:**

Estudo transversal desenvolvido num ambulatório na China.

**MÉTODOS:**

Pacientes pediátricos com amigdalite crônica foram recrutados. A infecção por *H.* *pylori* foi determinada segundo a presença de anticorpos *H. pylori* CagA IgG. Foi determinado o nível de cotinina sérica e exposição à FAT de todos os participantes.

**RESULTADOS:**

Não houve diferença significativa entre crianças com amigdalite crônica na infecção por *H.* *pylori* e sem amidalite, mas existia diferença significativa na FAT entre os dois grupos (P = 0,011). Em seguida, estudamos a associação entre FAT e amigdalite crônica com base no *status* de infecção por *H.* *pylori*. Nos pacientes com infecção por *H.* *pylori*, houve diferença significativa na distribuição de FAT entre os grupos de amigdalite crônica e controle (P = 0,022). Tomando os participantes sem FAT como referência, a análise de regressão logística multivariada mostrou que aqueles com alta FAT tinha maior susceptibilidade à amigdalite crônica (OR ajustado IC = 2,33, 95%: 1,67-3,25, ajustado P < 0,001). No entanto, naqueles sem infecção por *H.* *pylori*, a FAT não predispôs a amigdalite crônica.

**CONCLUSÃO:**

Nossos achados sugerem que a exposição ao tabaco é um fator de risco para amigdalite crônica em crianças com infecção por *H.* *pylori*.

## INTRODUCTION

*Helicobacter pylori* (*H. pylori*) is a chronic infectious pathogen with high prevalence. The *H. pylori* infection rate is as high as 70% in developing countries.^1^ It commonly occurs in children before the age of 10 years and even as early as 6 years in some countries.^2^ Typically, *H. pylori* infects the stomach, and has been associated with gastritis, peptic ulcer disease, gastric cancer and gastric mucosa-associated lymphoma in humans. *H. pylori* infection may also participate in some non-digestive diseases, such as nutritional iron deficiency anemia, growth retardation, malnutrition, autoimmune idiopathic thrombocytopenic purpura and chronic urticaria in children, as well as the development of adult atherosclerosis-related cardiovascular diseases and some nervous system diseases.^3^^,^^4^^,^^5^^,^^6^^,^^7^ Recently, several studies reported *H. pylori* colonization in locations outside the gastrointestinal cavity, such as adenotonsillar tissues and nasal and sinus mucosa.^3^^,^^8^

Chronic tonsillitis is one of the most frequent otolaryngological diseases in children. It causes symptoms that include poor appetite, sleep disorders, snoring, dysphagia and even growth retardation.^9^^,^^10^ The role of *H. pylori* infection in chronic tonsillitis remains controversial. Previous studies did not find evidence supporting *H. pylori* colonization of tonsillar tissues in the setting of chronic tonsillitis.^2^^,^^11^ A systematic review and meta-analysis showed that there was no significant difference in tonsillar *H. pylori* colonization between tissue samples derived from secondary to recurrent tonsillitis and samples from control children. Thus, those analyses did not provide any evidence that *H. pylori* infection might play a role in the pathogenesis or development of chronic tonsillitis.^11^ However, a very recent study reported that *H. pylori* was present in the tonsillar tissues of patients with chronic tonsillitis, using the Scorpion real-time polymerase chain reaction (PCR).^12^ Using a rapid urease test, another report showed that *H. pylori* was present in 30.5% of the tonsillar tissue of patients with chronic recurrent tonsillitis.^13^

The deleterious effects of environmental tobacco smoke (ETS) exposure on the upper respiratory tract of children are becoming increasingly recognized. A previous study showed that there was a significant association between children’s sore throats and maternal smoking. A retrospective case-control study showed that nearly half of children who underwent tonsillectomy to treat recurrent tonsillitis had previous smoke exposure. Further analysis indicated that children with ETS exposure had more than twice the odds of undergoing tonsillectomy for recurrent tonsillitis, compared with those without smoke exposure.^14^ Another study revealed the deleterious effects of parental smoking on upper respiratory tract infections in their children. A marked and statistically significant association was found between the incidence of tonsillectomy in children and parental smoking in the home environment. There was a higher frequency of attacks of tonsillitis requiring antibiotic treatment among the children whose parents smoked. If parents stopped smoking, the incidence of tonsillitis and the need for tonsillectomy in their children were diminished.^15^

So far, it remains unknown whether smoke exposure influences the role of *H. pylori* in tonsillitis in children.

## OBJECTIVE

We aimed to investigate the interaction between environmental tobacco exposure and *H. pylori* infection regarding the incidence of chronic tonsillitis among Chinese children. 

## METHODS

This was a cross-sectional study performed in an outpatient clinic in China.

The subjects of this study were child patients (2.5 to 14 years of age) with chronic tonsillitis who were admitted to the hospital affiliated to Binzhou Medical University for tonsillectomy between May 2012 and May 2014. Chronic tonsillitis was defined clinically as chronic infection of the palatine tonsils, on the basis of recurrent tonsillitis. None of the participants were smokers. We obtained information about environmental tobacco smoke exposure through questionnaires applied to each participant’s parents, adult household members and regular visitors. We obtained information about the smoking status of each participant’s parents, adult household members and regular visitors. We counted the intensity of environmental tobacco exposure in terms of the number of cigarettes consumed daily.

Meanwhile, we also recruited age and sex-matched healthy children who had annual check-ups at our hospital between May 2012 and May 2014. Questionnaires were also answered by the controls’ parents, and only individuals without self-reported ETS exposure were enrolled as controls.

This study was conducted in accordance with the principles expressed in the Declaration of Helsinki. All participants or their legal guardian gave their written informed consent, and the study protocol was approved by the Institutional Review Board of Binzhou Medical University (BMU-245).

### Blood sampling and serum cotinine analysis

Blood samples were collected from the participants and serum was obtained from them through centrifugation. Serum cotinine levels were quantified by using an enzyme-linked immunosorbent assay (ELISA; Cosmic Corporation, Tokyo, Japan) that had a detection limit of 0.6 ng/ml and an inter-assay variation of < 7%. The mean serum cotinine level in the subjects with ETS was 3.76 ± 0.21 ng/ml.

### Detection of H. pylori CagA IgG Antibodies

Blood samples were collected from all participants at enrollment and serum was isolated by means of centrifugation. *H. pyl*ori CagA IgG antibodies were detected in the patients’ serum using ELISA kits (MyBioSource, San Diego, CA, USA) in accordance with the manufacturer’s instructions. Samples with an antibody index greater than 0.9 were considered positive.^16^

### Statistical analysis

Differences in demographic characteristics between patients and controls were compared by using Student’s t test for continuous variables and the χ^2^ test or Fisher’s exact test for categorical variables. Based on *H. pylori* infection status, all the participants were allocated to subgroups and multiple logistic regression analyses with adjustment for age, sex, body weight, height and education level were performed to determine the risk factors for chronic tonsillitis among the patients. A forward stepwise (likelihood ratio) procedure was used for multivariable analysis. The data were analyzed using the SPSS 13.0 package (SPSS, Inc.) and the results were considered statistically significant at P < 0.01 using a two-tailed test. P-values < 0.05 were considered statistically significant.

## RESULTS

There were no significant differences in the distribution of age, sex, height, weight, education or place of residence between the patients and the healthy controls (Table 1; P > 0.05). Regarding the prevalence of *H. pylori* infection among the participants, 81.6% (186 out of the total of 228 patients) were positive for *H. pylori* infection and 83.2% were positive in the control group (199 out of the total of 239 patients). Overall, there was no significant difference in *H. pylori* infection prevalence in the children with chronic tonsillitis, compared with subjects free from this condition. (P = 0.632) (Table 2).

**Table 1:**
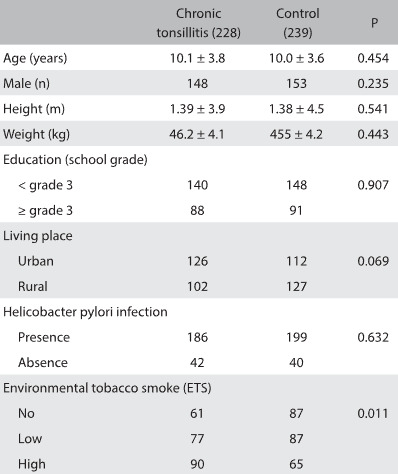
Clinical characteristics of the chronic tonsillitis and control groups

ETS = environmental tobacco smoke.

**Table 2:**
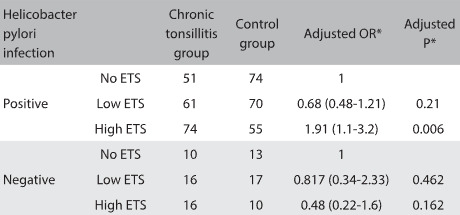
Association between ETS and chronic tonsillitis based on *Helicobacter pylori* infection status

ETS = environmental tobacco smoke; OR = odds ratio. *adjusted for confounding factors, including age, sex distribution, body weight, height and education level.

There were 148 participants (61 children with tonsillitis and 87 free of this condition) with no ETS (0 cigarettes/day). The other 319 presented ETS and their mean serum cotinine level was 3.76 ± 0.21 ng/ml. Using this mean serum cotinine value as the cutoff value, these participants were categorized as presenting either low ETS (less than 3.76 ng/ml; n = 77 in the chronic tonsillitis group and n = 87 in the controls) or high ETS (greater than or equal to 3.76 ng/ml; n = 90 in the chronic tonsillitis group and n = 65 participants without tonsillitis). Overall, there was a significant difference in ETS between the children with and without chronic tonsillitis (P = 0.011).

We next studied the association between ETS and chronic tonsillitis based on the *H. pylori* infection status. Among the individuals with *H. pylori* infection, there were 51 without ETS, 61 with low-level ETS and 74 with high-level ETS, while among the children without tonsillitis, there were 74 without ETS, 70 with low ETS and 55 with high ETS. There was a significant difference in ETS distribution between the two groups (P = 0.022). Taking the participants without ETS as the reference, multivariate logistic regression analysis showed that those with high ETS had higher susceptibility to chronic tonsillitis (adjusted OR = 2.33; 95% CI: 1.67-3.25; adjusted P < 0.001), with adjustment for age, sex, body weight, height and education level. However, among those without *H. pylori* infection, the ETS distribution was similar between the two groups (P = 0.415).

## DISCUSSION

This study provides the first report on an association of *H. pylori* infection and environmental tobacco exposure with the incidence of chronic tonsillitis in Chinese children. Our findings suggest that tobacco exposure is a risk factor for chronic tonsillitis among children with *H. pylori* infection. Therefore, it is important to have a tobacco-free environment for children who are subject to *H. pylori* infection.

*H. pylori* bacteria can release virulence factors, including the outer inflammatory protein produced by cytotoxin-associated gene A (CagA), which disrupts cell polarity, promotes apoptosis of epithelial cells and inhibits T cell proliferation in the gastric mucosa and upper respiratory tract.^17^*H. pylori* is detectable in tonsillar tissues and viable *H. pylori* can colonize these tissues. *H. pylori* has been identified in both tonsillar surface and core tissues.^17^ A histopathological assessment of tonsillar tissues found that 130 (39.6%) out of 285 children were positive for *H. pylori* and that the rapid urease test was not sensitive enough as a diagnostic tool. A recent review regarding *H. pylori* colonization and chronic tonsillitis showed that *H. pylori* colonization was not more prevalent in tonsillar tissue with chronic or recurrent infections.^11^ In our study, we used the PCR method to detect CagA IgG, in order to determine *H. pylori* infection. Consistent with the abovementioned reports, our data showed that the *H. pylori* infection rates were not significantly different between children with and without chronic tonsillitis.

An association between passive smoking and *H. pylori* infection was reported in a study conducted in Germany, which investigated the relationship between parental smoking and *H. pylori* infection in a population-based study among preschool children. After adjustment for confounding factors, a strong positive relation between smoking by the father in the household and *H. pylori* infection (odds ratio = 3.7; 95% confidence interval = 2.3-6.1).^18^^,^^19^ Cirak et al. demonstrated a relatively high rate of *H. pylori* infection in adenotonsillectomy specimens, through using PCR to detect the CagA gene. They postulated that the tonsil and adenoid tissue may be an ecological niche within the mouth.^20^ Likewise, we also detected high rates of *H. pylori*-positive findings using a similar PCR method (81.5% in chronic tonsillitis patients and 83.2% in controls). Di Bonaventura et al. were unable to detect *H. pylori* by means of PCR on tonsil swabs and biopsy materials from their patients, although *H. pylori* was detected in gastric biopsy cultures. They suggested that the tonsils are not an extragastric reservoir for H. pylori infection.^2^ Neither of those studies took smoking status into account.

In our study, none of the participants were smokers. Nonetheless, a considerable proportion presented environmental tobacco exposure. A very early study revealed a marked and statistically significant association between the incidence of tonsillectomy among children and parental smoking in the home environment.^15^ There was a higher frequency of attacks of tonsillitis requiring antibiotic treatment among the children whose parents smoked.^15^ Among children who underwent tonsillectomy due to recurrent tonsillitis, 47.27% had previously been subject to smoke exposure, compared with 67 (27.80%) in the hernia repair group. Logistic regression indicated that children with smoke exposure had more than twice the odds of undergoing tonsillectomy due to recurrent tonsillitis, compared with those with no exposure. In our study, we found that the majority of the participants (73.2% of the chronic tonsillitis patients and 63.6% without this condition) were exposed to environmental smoke (77 with low ETS and 90 with high ETS among the chronic tonsillitis patients; 87 with low ETS and 65 with high ETS among the controls). These high ETS exposure rates suggest that there is an urgent need for a tobacco-free environment for Chinese children. Similarly, our data also show that among those with H. pylori, the risk of chronic tonsillitis was nearly twice the risk among those without it.

Several limitations to this study should be noted. Firstly, we only used the *H. pylori* CagA IgG antibody detection method to detect *H. pylori* infection. Secondly, with 298 participants, the sample size was relatively small. Thirdly, the exact molecular mechanism under which environmental tobacco exposure and *H. pylori* infection predispose towards chronic tonsillitis was not studied.

## CONCLUSION

In this study, we reported the interaction between environmental tobacco smoke exposure and *H. pylori* infection for increasing susceptibility towards chronic tonsillitis. This finding suggests that it is important to stop environmental tobacco smoke exposure among children in order to reduce the risk of chronic tonsillitis among children.
